# Soluble epoxide hydrolase (sEH) is a novel anti‐inflammatory target and a first‐in‐class, picomolar sEH inhibitor (EC5026) improves cognition in a mouse model of Alzheimer’s disease

**DOI:** 10.1002/alz.089528

**Published:** 2025-01-09

**Authors:** Yichen Li, Yuan Hou, Cindy McReynolds, Andrew A. Pieper, James B Leverenz, Jeffrey L. Cummings, Bruce D. Hammock, Feixiong Cheng

**Affiliations:** ^1^ Cleveland Clinic, Cleveland, OH USA; ^2^ Eicosis Inc., Davis, CA USA; ^3^ Case Western Reserve University, Cleveland, OH USA; ^4^ 1950 East 89th Street, 1950 East 89th Street, OH USA; ^5^ Cleveland Clinic Lou Ruvo Center for Brain Health, Cleveland, OH USA; ^6^ Chambers‐Grundy Center for Transformative Neuroscience, Department of Brain Health, School of Integrated Health Sciences, University of Nevada Las Vegas, Las Vegas, NV USA; ^7^ University of California, Davis, Davis, CA USA

## Abstract

**Background:**

Although investment in biomedical and pharmaceutical research has increased significantly over the past two decades, there are no oral disease‐modifying treatments for Alzheimer’s disease (AD).

**Method:**

We performed comprehensive human genetic and multi‐omics data analyses to test likely causal relationship between EPHX2 (encoding soluble epoxide hydrolase [sEH]) and risk of AD. Next, we tested the effect of the oral administration of EC5026 (a first‐in‐class, picomolar sEH inhibitor) in a transgenic mouse model of AD‐5xFAD and mechanistic pathways of EC5026 in patient induced Pluripotent Stem Cells (iPSC) derived neurons. 5xFAD mice were treated with 3 mg/kg EC5026 in drinking water for 3 months starting at 3 months of age. We evaluated the effect of EC5026 treatment on cognition using the novel object recognition test, Morris water maze and a fear conditioning test.

**Result:**

We identified elevated sEH protein level (protein expression quantitative trait loci [pQTL] from the AD knowledge portal) is significantly associated with increased AD risk across all five large‐scale AD genome‐wide association studies (GWAS). A genome‐wide significant, protein‐coding SNP rs2741342 on *EPHX2* is replicated in three pQTL datasets from the ROSMAP biobank. Further genetic and eQTL/pQTL co‐localization and fine‐mapping analyses confirmed that EPHX2 was a causal target in AD. We found that EC5026 reduced tau hyper‐phosphorylation (pTau205) in AD patient iPSC‐derived neurons. The 3 months oral administration of EC5026 significantly improved the cognition of 5xFAD transgenic mice on novel object recognition, Morris water maze and a contextual fear conditioning test. Bulk and single‐cell RNA‐sequencing analysis of EC5026‐treated brain sections revealed potential anti‐inflammatory mechanistic pathways and brain target engagement to be tested in future clinical trials.

**Conclusion:**

In summary, we demonstrate that EPHX2 (sEH) is a promising novel anti‐inflammatory target for AD and the EC5026 (a first‐in‐class, picomolar sEH inhibitor) significantly improves cognition in the 5xFAD mouse model. Future functional observations and clinical trials are warranted to validate the causal relationship of EC5026 in potential treatment of AD.

